# Prevalence of burnout syndrome among unmatched trainees and residents in surgical and nonsurgical specialties: a cross-sectional study from different training centers in Palestine

**DOI:** 10.1186/s12909-022-03386-8

**Published:** 2022-04-26

**Authors:** Ramzi Shawahna, Iyad Maqboul, Ola Ahmad, Afnan Al-Issawy, Batoul Abed

**Affiliations:** 1 Department of Physiology, Pharmacology and Toxicology, Faculty of Medicine and Health Sciences, An-Najah National University, Nablus, Palestine; 2 An-Najah BioSciences Unit, Centre for Poisons Control, Chemical and Biological Analyses, An-Najah National University, Nablus, Palestine; 3 Department of Medicine, Faculty of Medicine & Health Sciences, An-Najah National University, P.O. Box 7, Nablus, Palestine; 4 An-Najah National University Hospital, Nablus, Palestine

**Keywords:** Burnout, Copenhagen Burnout Inventory, Medical education, Training, Resident, Palestine

## Abstract

**Background:**

Burnout is a psychological syndrome that involves physical, mental, and emotional exhaustion. This study was conducted to determine the prevalence of burnout among unmatched trainees and residents in surgical and nonsurgical specialties in Palestine. Additionally, this study also sought to identify the associated variables and predictors of higher burnout scores.

**Methods:**

This study was conducted in a cross-sectional design using a questionnaire in the period between October 2020 and March 2021. The questionnaire collected sociodemographic characteristics of the residents and trainees in 18 different training centers/hospitals. The Copenhagen Burnout Inventory was used to determine burnout among the residents and trainees. Pearson’s correlations, analysis of variance, Student’s t-test, and multiple linear regressions were used to analyze the data.

**Results:**

The study tool was completed by 250 residents and trainees in surgical and nonsurgical specialties (response rate = 83.3%). Of all participants, 203 (81.2%) reported a moderate-severe degree of burnout in the personal domain, 188 (75.2%) reported a moderate-severe degree of burnout in the work-related domain, and 97 (38.8%) reported moderate-severe degree of burnout in the client-related domain. There was a moderate and positive correlation between personal and work-related burnout scores (Pearson’s r = 0.69, p-value < 0.001), and client-related burnout scores (Pearson’s r = 0.52, p-value < 0.001). Similarly, there was a moderate and positive correlation between work-related and client-related burnout scores (Pearson’s r = 0.57, p-value < 0.001). The multiple linear regression model showed that higher burnout scores were predicted by being married, not having another paid employment, inability to financially support oneself, frequent consumption of tea/coffee, dissatisfied with the training/job, thinking to change the profession, and long working hours.

**Conclusion:**

The findings of this study indicated that burnout was highly prevalent among residents and trainees in surgical and nonsurgical specialties in Palestine. Decision-makers in healthcare authorities, hospital managers, professional groups, directors of residency programs, and educators/trainers should consider addressing burnout and improving the well-being of residents and trainees in surgical and nonsurgical specialties in Palestine. Future studies are still needed to determine which interventions could be effective in reducing burnout among residents and trainees in surgical and nonsurgical specialties in Palestine.

**Supplementary Information:**

The online version contains supplementary material available at 10.1186/s12909-022-03386-8.

## Background

Burnout is a psychological syndrome that involves physical, mental, and emotional exhaustion [1]. The syndrome is believed to arise from responses to persistent interpersonal stress factors that can be personal, work, and/or client-related [2–5]. Signs and symptoms of burnout might also include a sense of depersonalization and dissatisfaction with personal accomplishment. The psychological symptoms of burnout include irritability, cynicism, and decreased concentration [6].

Burnout was reported as a major health issue among physicians and residents in different specialties [5, 7, 8]. A recent meta-analysis reported high prevalence rates of burnout among residents in radiology, neurology, and general surgery that were estimated at (77.16%, 95% CI: 5.99%–99.45%), (71.93%, 95% CI: 65.78%–77.39%), and (58.39%, 95% CI: 45.72%–70.04%), respectively [7]. Previous studies have identified several factors that could promote burnout among physicians and residents. Women were reported to experience burnout at a higher rate than their male counterparts [9]. Additionally, higher levels of burnout were associated with long working hours, lack of respect from colleagues, increasing computerization of practice, insufficient compensation, lack of clinical autonomy, and lack of time for social life [1, 10, 11]. In a qualitative study, residents have reported that being a doctor superseded them [12]. Additionally, the residents reported an inability to balance their work and personal lives which affected their well-being [12].

It has been argued that supportive personal relationships helped residents improve their well-being and protected them against depression [1]. Physicians also reported some particular workplaces or situations were especially stressful [9]. These included all types of situations associated with isolation, directly facing death, risk-taking, and having too much empathy for patients and their relatives. Personal factors including the burden of personal responsibility, feelings of guilt or helplessness, and doubts about their abilities also contributed to burnout [1]. Previous studies have also shown that physicians who favored their academic success, who were perfectionists, who felt compulsiveness, guilt, and self-denial were more susceptible to burnout [13, 14].

Burnout was shown to affect the ability of physicians to provide compassionate and centered care to their patients [15]. Consequently, burnout can increase the risk of alcohol and/or drug abuse, motor vehicle accidents, and relationship stress [16]. These might consequently increase the risk of committing medical errors, impair professionalism, reduce the productivity of physicians, and increase the risk of sick leaves [17, 18]. In a recent study, about 14% of the physicians who reported burnout also reported having suicidal thoughts [10]. Of those, only one-third sought treatment. Lack of treatment-seeking behavior was attributed to having limited time to seek medical care, the stigma associated with treatment for psychological illnesses, and fear of career turnovers [19–21].

Specialty training is academically challenging, mentally, and physically demanding. Specialty training was shown to be associated with high rates of stress during residency training and later practice. Burnout and depression were reported at higher rates among surgery residents [7, 22]. A previous study showed that 6% of surgeons reported suicidal ideations in the prior 12 months [23]. Of those, only 26% had sought help due to concerns about how seeking treatment could affect their license to practice [23].

In Palestine, specialty training can be very stressful. A previous study showed that depressive and anxiety symptoms were highly prevalent among Palestinian medical students [24]. In Palestine, graduates of the Doctor of Medicine (MD) programs are required to complete a 1-year training before they can appear in an examination that is held by the Palestine Medical Council to qualify for surgical and nonsurgical residency programs. After passing the examination, qualified candidates can seek opportunities to match in one of the available residency programs. Residents are required to complete rotations in different wards and units. On average, residents complete their residency programs in 4 to 5 years. During this period, residents have to work long shift hours and provide care to a large number of patients. Like in many countries, residents receive low payments during their residency programs. Assessing the prevalence of burnout and identifying stressors among unmatched trainees and residents in surgical and nonsurgical specialties could help guide interventions to address these stressors and improve the well-being of this important segment of highly skilled healthcare professionals [25, 26]. Currently, little is known on the prevalence of burnout among unmatched trainees and residents in surgical and nonsurgical specialties in Palestine. Therefore, this study was conducted to determine the prevalence of burnout among residents and trainees in surgical and nonsurgical specialties in Palestine. Additionally, this study also sought to identify the associated variables and predictors of higher burnout scores.

## Methods

### Study design 

This study was conducted in a cross-sectional design in the period between October 2020 and March 2021 to assess the prevalence of burnout among unmatched trainees and residents in surgical and nonsurgical specialties in the context of understanding the factors associated with higher burnout. The study was conducted and reported in compliance with the guidelines for reporting cross-sectional studies in which a questionnaire was used as the study tool [27–29]. Compliance with the checklist is shown in Supplementary Table S1. The checklist ensured reporting study design, objectives, settings, recruitment process, sampling, participants, representativeness, study tool, reliability/validity, psychometrics, data analysis, main results, interpretation of the findings, discussion of the strengths, limitations, and generalizability.

### Study settings 

Holders of MD degrees could have graduated from one of the 2 medical schools in Palestine (An-Najah National University or Al-Quds University) or could have obtained their degrees from a medical school abroad. To qualify for a training/residency program in Palestine, MD graduates have to pass an examination that is organized by the Palestine Medical Council. Based on the results of the examination, the candidates qualify for one of the two tracks: surgical or non-surgical specialties. According to the matching criteria of the Palestine Medical Council, surgical specialties include general surgery, gynecology and obstetrics, orthopedic surgery, urologic surgery, surgical ophthalmology, surgical otolaryngology, cardiac surgery, and neurosurgery. On the other hand, nonsurgical specialties include internal medicine, family medicine, dermatology, pediatrics, neonatology, radiology, psychiatry, cardiology, pulmonology, oncology, anesthesia and intensive care, rheumatology, nephrology, hematology, and pathology. At the time of the study, the residency/training programs in Palestine were offered by 18 designated centers/hospitals across the West Bank of Palestine. These centers/hospitals were: An-Najah National University Hospital, Al-Watani Medical Hospital, Jenin Governmental Hospital, Specialized Arab Hospital, Makassed Hospital, Palestine Medical Complex, Augusta Victoria Hospital, Al-Istishari Arab Hospital, Al-Ahli Hospital, Hebron Governmental Hospital, Rafidia Surgical Hospital, Tulkarm Governmental Hospital, Saint Joseph Hospital, Red Crescent Society Hospital, Holy Family Hospital, Caritas Hospital for Children, Beit Jala Governmental Hospital, and Saint John Eye Hospital. The distribution of these training centers/hospitals across the West Bank is shown in Supplementary Figure S1. This study was conducted among unmatched trainees and residents in surgical and nonsurgical specialties in the Palestinian hospitals with active residency programs.

### Study population and sample size

In this project, the study population was unmatched trainees and residents in surgical and nonsurgical specialties in Palestinian practice. To compute the sample size needed for this study, an online sample size calculator was used. The sample size calculator can be freely accessed at (www.raosoft.com/samplesize.html). The sample size needed for this study was calculated for a pooled population of 750 unmatched trainees and residents in the 18 training centers/hospitals at a 95% confidence interval (CI) and tolerating a margin of error of 5%. The sample size needed for this study was 255. A convenience sample approach was used to recruit the participants in this study.

Unmatched trainees and residents in surgical and nonsurgical specialties were eligible for inclusion in this study when they fulfilled the following criteria: a) completed a graduating degree in medicine, b) completed or were in a house job/training period, c) fulfilled the eligibility requirements for the surgical or nonsurgical residency program, d) expressed willingness to respond to the questionnaire, and e) provided informed consent.

### Study tool

The study tool used in this study was a questionnaire. The questionnaire contained 2 parts. The first part collected the sociodemographic and practice details of the trainees and residents. In this part, the trainees and residents in surgical and nonsurgical specialties were asked to provide their gender, marital status, if they had children, if they lived with parents, if they had another paid employment if they were able to support themselves financially, if they financially supported self, wife, children, parents, if they owned a car, if they owned a laptop, if they owned a smartphone, duration of their sleep per 24 h, smoking status, number of tea/coffee cups consumed per day, if they thought of changing their profession, if they thought of changing their specialty, if they were satisfied with their training/job, number of hours/week, and the single longest duty hours. The first part of the questionnaire is provided in Supplementary Table S2. In the second part, the questionnaire contained the Copenhagen Burnout Inventory [30]. The Copenhagen Burnout Inventory was developed as a tool to assess burnout among professionals including physicians, nurses, midwives, and other healthcare professionals. The inventory contained 19-items to measure personal burnout (6 items), work-related burnout (7 items), and client-related burnout (6 items). On each item, the participants had to respond by either always/to a very high degree, often/to a high degree, sometimes/somewhat, seldom/to a low degree, never/seldom/to a very low degree.

The inventory was previously validated and the Cronbach’s alpha for the personal burnout items was 0.87, work-related burnout was 0.87, and client-related burnout was 0.85 [30].

In this study, the questionnaire was pilot tested with 20 final-year medical students who completed their rotations/training. To assess the reliability of scores over a short time, the test–retest method was used. The medical students were asked to respond to the questionnaire. After 1–2 h, the same students were asked to respond to the questionnaire again. Scores obtained in the 2 rounds were correlated using Pearson’s correlation. It was decided a priori that a Pearson’s r of ≥ 0.8 would indicate acceptable stability of scores over a short time. In this study, Pearson’s r was 0.96 which indicated excellent stability of scores over a short time. The internal consistency of the inventory was assessed using Cronbach’s alpha. It was decided a priori that a Cronbach’s alpha of 0.70 indicated acceptable internal consistency of the items. In this study, the personal burnout items had a Cronbach’s alpha of 0.89, the work-related burnout items had a Cronbach’s alpha of 0.80, and the client-related burnout items had a Cronbach’s alpha of 0.70. The 19-item inventory had a Cronbach’s alpha of 0.84.

### Data collection

The study participants were visited in the training centers/hospitals by the field researchers who were final-year medical students. The field researchers explained the objectives of the study to the potential participants and invited them to respond to the questionnaire. The participants were informed that this study was voluntary and was asked to provide written informed consent before they responded to the questionnaire. The participants did not receive any incentives in exchange for their participation in this study.

### Statistical analysis

Responses of the participants were covered into scores as follows [30]: a) always/to a very high degree = 100%, b) often/to a high degree = 75%, c) sometimes/somewhat = 50%, d) seldom/to a low degree = 25%, and e) never/almost never/to a very low degree = 0%. The burnout categories were assigned as follows [31]: a) score < 50, no/low burnout, b) 74 ≥ score ≥ 50, moderate burnout, c) 99 ≥ score ≥ 75, and d) score = 100, severe burnout.

The data were entered into IBM SPSS for Windows v. 21.0. The data were tested for normality of distribution using the Kolmogorov–Smirnov test. Data were expressed as means with their corresponding standard deviations (SD). As the data were normally distributed, parametric tests were used to analyze the data. Groups were compared using either analysis of variance (ANOVA) or Student’s t-test, as appropriate. Correlations were investigated using Pearson’s correlations. Multiple linear regression was used to control confounding variables and identify predictors of higher burnout scores. In this study, the variables with a p-value of < 0.25 in the ANOVA or Student’s t-test were retained and entered into the multiple linear regression models. Goodness-of-fit was indicated by the adjusted R-squared values when the p-value was < 0.05. Multicollinearities were assessed using tolerance and variance inflation factor (VIF) values. Tolerance values of > 0.1 and VIF values of close to 1 indicated the absence of multicollinearity issues. A p-value of < 0.05 indicates statistical significance.

### Ethics approval and consent to participate

The study was conducted in adherence to the regulations and ethics followed at An-Najah National University and in compliance with the Declaration of Helsinki. The study received ethical approval from the Institutional Review Board (IRB) of An-Najah National University. Each participant provided written informed consent before taking part in the study.

## Results

### Response rate and characteristics of the participants

In this study, 300 unmatched trainees and residents in surgical and nonsurgical specialties were invited. Of those, 250 completed the study tool, giving a response rate of 83.3%. Of the respondents, 135 (54.0%) were male, 147 (58.8%) were single, 181 (72.4%) had no children, and 150 (60.0%) lived with parents. Of all participants, 37 (14.8%) had another paid employment, 179 (71.6%) were able to financially support themselves, 107 (42.8%) financially supported wife/children/parents, 87 (34.8%) owned a car, 197 (78.8%) owned a laptop, 241 (96.4) owned a smartphone. In this study, 143 (57.2%) slept less than 6 h/24 h, 82 (32.8%) were smokers, 171 (68.4%) consumed 2 or more cups of tea/coffee per day. Of the participants, 195 (78.0%) thought of changing their profession, 167 (66.8%) thought of changing their specialty, 74 (29.6%) were not satisfied with their training/job, 143 (57.2%) worked 60 and more hours per week, and 200 (80.0%) reported a single duty of 24 h and more. Details of the responses of the participants are shown in Table 1.

**Table 1:** Sociodemographic characteristics of the participants (*n* = *250*)

Variable	n	%
**Current training/residency status**		
Unmatched trainee	113	45.2
Resident in a surgical specialty	71	28.4
Resident in a nonsurgical specialty	66	26.4
**Gender**		
Male	135	54.0
Female	115	46.0
**Marital status**		
Single	147	58.8
Married	103	41.2
**Have children**		
No	181	72.4
Yes	69	27.6
**Living with parents**		
No	100	40.0
Yes	150	60.0
**Have another paid employment**		
No	213	85.2
Yes	37	14.8
**Able to support yourself financially**		
No	71	28.4
Yes	179	71.6
**Financially supporting**		
Self	143	57.2
Wife/children/parents	107	42.8
**Own a car**		
No	163	65.2
Yes	87	34.8
**Own a laptop**		
No	53	21.2
Yes	197	78.8
**Own a smartphone**		
No	9	3.6
Yes	241	96.4
**Sleep duration (hours) per 24 h**		
< 6	143	57.2
≥ 6	107	42.8
**Smoking**		
No	168	67.2
Yes	82	32.8
**Consumption of tea/coffee**		
Not frequent (up to 1 cup per day)	79	31.6
Frequent (≥ 2 cup per day)	171	68.4
**Thought of changing your profession**		
No	55	22.0
Yes	195	78.0
**Thought of changing your specialty**		
No	83	33.2
Yes	167	66.8
**Satisfied with your training/job**		
No	74	29.6
Yes	176	70.4
**Number of working hours per week**		
< 60	107	42.8
≥ 60	143	57.2
**Single longest duty hours**		
< 24	50	20.0
≥ 24	200	80.0

### Prevalence of burnout 

Of all participants, 203 (81.2%) reported a moderate-severe degree of burnout on the personal domain, 188 (75.2%) reported a moderate-severe degree of burnout on the work-related domain, and 97 (38.8%) reported moderate-severe degree of burnout on the client-related domain. Details of the prevalence of burnout among the participants are shown in Table 2.

**Table 2:** Prevalence of burnout symptoms among the study participants (*n* = *250*)

Domain	Category	n	%
Personal	No/low burnout	47	18.8
	Moderate burnout	139	55.6
	High burnout	58	23.2
	Severe burnout	6	2.4
Work-related	No/low burnout	62	24.8
	Moderate burnout	135	54.0
	High burnout	52	20.8
	Severe burnout	1	0.4
Client-related	No/low burnout	153	61.2
	Moderate burnout	86	34.4
	High burnout	11	4.4
	Severe burnout	0	0.0

### Associations between burnout scores and variables of the participants

In this study, there was a moderate and positive correlation between personal and work-related burnout scores (Pearson’s r = 0.69, p-value < 0.001), and client-related burnout scores (Pearson’s r = 0.52, p-value < 0.001). Similarly, there was a moderate and positive correlation between work-related and client-related burnout scores (Pearson’s r = 0.57, p-value < 0.001).

Personal burnout scores were not statistically significant between unmatched trainees, residents in surgical specialties, and residents in nonsurgical specialties. On the other hand, the mean personal burnout scores were significantly higher for residents, married, not living with parents, not having another paid employment, financially supporting the family (wife/children/parents), not owning a laptop, sleeping for less than 6 h, smokers, frequently consuming tea/coffee, thought of changing profession, thought of changing specialty, not satisfied with their job/training, worked for 60 and more hours, and worked for a single duty of 24 h and more. Details of the associations are shown in Table 3.

**Table 3:** Association between variables of the participants and personal burnout scores

Variable	n	%	Mean	SD	*P*-value
**Current training/residency status**					
Unmatched trainee	113	45.2	59.8	16.3	0.110
Resident in surgical specialty	71	28.4	64.0	15.0	
Resident in nonsurgical specialty	66	26.4	64.3	16.4	
**Gender**					
Male	135	54.0	61.9	16.1	0.662
Female	115	46.0	62.5	16.1	
**Marital status**					
Single	147	58.8	59.6	15.3	0.001
Married	103	41.2	65.9	16.3	
**Have children**					
No	181	72.4	61.4	15.9	0.147
Yes	69	27.6	64.2	16.4	
**Living with parents**					
No	100	40.0	65.3	16.6	0.005
Yes	150	60.0	60.1	15.3	
**Have another paid employment**					
No	213	85.2	63.4	15.6	0.008
Yes	37	14.8	55.5	17.2	
**Able to support yourself financially**					
No	71	28.4	63.4	16.1	0.408
Yes	179	71.6	61.7	16.0	
**Financially supporting**					
Self	143	57.2	60.2	15.3	0.013
Wife/children/parents	107	42.8	64.8	16.7	
**Own a car**					
No	163	65.2	62.0	15.9	0.594
Yes	87	34.8	62.5	16.4	
**Own a laptop**					
No	53	21.2	65.8	14.8	0.040
Yes	197	78.8	61.2	16.2	
**Own a smartphone**					
No	9	3.6	65.3	19.0	0.666
Yes	241	96.4	62.1	15.9	
**Sleep duration per 24 h**					
< 6	143	57.2	65.3	15.1	< 0.001
≥ 6	107	42.8	58.1	16.3	
**Smoking**					
No	168	67.2	60.6	16.1	0.018
Yes	82	32.8	65.5	15.5	
**Consumption of tea/coffee**					
Not frequent (up to 1 cup per day)	79	31.6	57.1	15.4	< 0.001
Frequent (≥ 2 cup per day)	171	68.4	64.6	15.8	
**Thought of changing your profession**					
No	55	22.0	54.6	16.2	< 0.001
Yes	195	78.0	64.3	15.4	
**Thought of changing your specialty**					
No	83	33.2	58.6	18.2	0.016
Yes	167	66.8	64.0	14.6	
**Satisfied with your training/job**					
No	74	29.6	68.7	13.5	< 0.001
Yes	176	70.4	59.4	16.2	
**Number of working hours per week**					
< 60	107	42.8	59.3	17.2	0.008
≥ 60	143	57.2	64.4	14.8	
**Single longest duty hours**					
< 24	50	20.0	56.8	17.0	0.002
≥ 24	200	80.0	63.6	15.5	

The work-related burnout scores were not statistically significant between unmatched trainees, residents in surgical specialties, and residents in nonsurgical specialties. On the other hand, the mean work-related burnout scores were significantly higher for married, not living with parents, not having another paid employment, financially supporting the family (wife/children/parents), sleeping for less than 6 h, smokers, frequently consuming tea/coffee, thought of changing profession, thought of changing specialty, not satisfied with their job/training, worked for 60 and more hours, and worked for a single duty of 24 h and more. Details of the associations are shown in Table 4.

**Table 4:** Association between variables of the participants and work-related burnout scores

Variable	n	%	Mean	SD	*p*-value
**Current training/residency status**					
Unmatched trainee	113	45.2	58.2	16.7	0.630
Resident in surgical specialty	71	28.4	60.2	15.0	
Resident in nonsurgical specialty	66	26.4	60.3	17.2	
**Gender**					
Male	135	54.0	58.5	17.2	0.450
Female	115	46.0	60.3	15.3	
**Marital status**					
Single	147	58.8	57.3	16.8	0.031
Married	103	41.2	62.2	15.4	
**Have children**					
No	181	72.4	58.7	16.9	0.417
Yes	69	27.6	60.9	14.8	
**Living with parents**					
No	100	40.0	61.8	16.1	0.053
Yes	150	60.0	57.6	16.3	
**Have another paid employment**					
No	213	85.2	60.6	15.9	0.004
Yes	37	14.8	51.7	17.1	
**Able to support yourself financially**					
No	71	28.4	63.2	15.6	0.011
Yes	179	71.6	57.8	16.4	
**Financially supporting**					
Self	143	57.2	58.2	15.7	0.215
Wife/children/parents	107	42.8	60.8	17.2	
**Own a car**					
No	163	65.2	59.1	16.4	0.976
Yes	87	34.8	59.7	16.3	
**Own a laptop**					
No	53	21.2	60.8	14.5	0.299
Yes	197	78.8	58.9	16.8	
**Own a smartphone**					
No	9	3.6	61.5	14.9	0.747
Yes	241	96.4	59.2	16.4	
**Sleep duration per 24 h**					
< 6	143	57.2	61.6	15.5	0.006
≥ 6	107	42.8	56.3	17.0	
**Smoking**					
No	168	67.2	58.2	16.3	0.055
Yes	82	32.8	61.5	16.3	
**Consumption of tea/coffee**					
Not frequent (up to 1 cup per day)	79	31.6	54.3	15.6	0.001
Frequent (≥ 2 cup per day)	171	68.4	61.6	16.2	
**Thought of changing your profession**					
No	55	22.0	51.0	17.7	< 0.001
Yes	195	78.0	61.6	15.2	
**Thought of changing your specialty**					
No	83	33.2	54.5	17.0	0.002
Yes	167	66.8	61.7	15.5	
**Satisfied with your training/job**					
No	74	29.6	66.0	16.6	< 0.001
Yes	176	70.4	56.5	15.4	
**Number of working hours per week**					
< 60	107	42.8	56.3	17.0	0.010
≥ 60	143	57.2	61.6	15.5	
**Single longest duty hours**					
< 24	50	20.0	54.2	17.7	0.014
≥ 24	200	80.0	60.6	15.8	

The client-related burnout scores were not statistically significant between unmatched trainees, residents in surgical specialties, and residents in nonsurgical specialties. On the other hand, the mean client-related burnout scores were significantly higher for married, not living with parents, having another paid employment, not able to financially support self, sleeping for less than 6 h, frequently consuming tea/coffee, thought of changing profession, thought of changing specialty, not satisfied with their job/training, worked for 60 and more hours, and worked for a single duty of 24 h and more. Details of the associations are shown in Table 5.

**Table 5:** Association between variables of the participants and client-related burnout scores

Variable	n	%	Mean	SD	p-value
**Current training/residency status**					
Unmatched trainee	113	45.2	42.8	16.4	0.119
Resident in surgical specialty	71	28.4	43.2	14.4	
Resident in nonsurgical specialty	66	26.4	47.9	18.4	
**Gender**					
Male	135	54.0	45.3	17.0	0.347
Female	115	46.0	43.0	15.9	
**Marital status**					
Single	147	58.8	41.4	15.8	0.002
Married	103	41.2	48.3	16.7	
**Have children**					
No	181	72.4	42.8	16.6	0.022
Yes	69	27.6	48.2	15.6	
**Living with parents**					
No	100	40.0	47.5	17.4	0.015
Yes	150	60.0	42.1	15.6	
**Have another paid employment**					
No	213	85.2	44.6	16.8	0.377
Yes	37	14.8	42.6	14.6	
**Able to support yourself financially**					
No	71	28.4	44.9	18.1	0.502
Yes	179	71.6	44.0	15.8	
**Financially supporting**					
Self	143	57.2	41.4	15.3	0.005
Wife/children/parents	107	42.8	48.1	17.3	
**Own a car**					
No	163	65.2	43.2	15.3	0.237
Yes	87	34.8	46.3	18.5	
**Own a laptop**					
No	53	21.2	45.4	15.3	0.814
Yes	197	78.8	44.0	16.8	
**Own a smartphone**					
No	9	3.6	50.9	13.6	0.201
Yes	241	96.4	44.0	16.6	
**Sleep duration per 24 h**					
< 6	143	57.2	46.6	16.0	0.014
≥ 6	107	42.8	41.2	16.7	
**Smoking**					
No	168	67.2	42.4	17.0	0.016
Yes	82	32.8	48.1	14.8	
**Consumption of tea/coffee**					
Not frequent (up to 1 cup per day)	79	31.6	39.5	14.7	0.001
Frequent (≥ 2 cup per day)	171	68.4	46.5	16.8	
**Thought of changing your profession**					
No	55	22.0	35.1	15.8	< 0.001
Yes	195	78.0	46.9	15.8	
**Thought of changing your specialty**					
No	83	33.2	39.3	17.3	0.001
Yes	167	66.8	46.7	15.6	
**Satisfied with your training/job**					
No	74	29.6	51.1	16.8	< 0.001
Yes	176	70.4	41.4	15.5	
**Number of working hours per week**					
< 60	107	42.8	40.8	16.6	0.009
≥ 60	143	57.2	46.9	16.0	
**Single longest duty hours**					
< 24	50	20.0	43.0	16.2	0.564
≥ 24	200	80.0	44.6	16.6	

### Predictors of burnout score 

Multiple linear regression models were used to identify significant predictors of high burnout scores. These predictors were arranged in order based on their standardized coefficients. The personal burnout scores were predicted by being married, dissatisfied with training/job, thinking of changing profession, not having another paid employment, and frequent consumption of tea/coffee. The work-related burnout scores were predicted by being dissatisfied with training/job, thinking of changing profession, being unable to financially support self, frequent consumption of tea/coffee, and not having another paid employment. Finally, the client-related burnout scores were predicted by being dissatisfied with training/job, thinking of changing profession, and working for 60 and more hours per week. Details of the predictors are shown in Table 6.

**Table 6:** Predictors of burnout score

Domain	Variable	Unstandardized Coefficients	SE	Standardized Coefficients	t	p-value
**Personal burnout**	Current training/residency status	-0.66	1.40	-0.03	-0.47	0.637
	Marital status	7.88	3.57	0.24	2.21	0.028
	Have children	-3.01	3.05	-0.08	-0.99	0.325
	Living with parents	-0.20	2.88	-0.01	-0.07	0.946
	Have another paid employment	-6.95	2.74	-0.15	-2.54	0.012
	Financially supporting self/others	2.03	2.25	0.06	0.90	0.367
	Able to support self financially	-1.30	2.17	-0.04	-0.60	0.550
	Own a laptop	-3.06	2.29	-0.08	-1.34	0.182
	Sleep duration per 24 h	-2.70	1.96	-0.08	-1.38	0.170
	Smoking	1.70	2.13	0.05	0.80	0.425
	Consumption of tea/coffee	5.17	2.08	0.15	2.49	0.014
	Thought of changing profession	6.83	2.60	0.18	2.62	0.009
	Thought of changing specialty	0.38	2.33	0.01	0.16	0.871
	Satisfied with training/job	-7.51	2.09	-0.21	-3.60	< 0.001
	Number of working hours	0.43	2.57	0.01	0.17	0.869
	Single longest duty hours	4.61	2.76	0.12	1.67	0.097
**Work-related burnout**	Marital status	5.79	3.65	0.17	1.58	0.115
	Have children	-1.03	3.13	-0.03	-0.33	0.743
	Living with parents	-1.12	2.95	-0.03	-0.38	0.704
	Have another paid employment	-6.50	2.81	-0.14	-2.31	0.022
	Financially supporting self/others	0.88	2.26	0.03	0.39	0.697
	Able to support self financially	-5.44	2.22	-0.15	-2.45	0.015
	Own a laptop	-0.35	2.34	-0.01	-0.15	0.882
	Sleep duration per 24 h	-0.74	2.01	-0.02	-0.37	0.714
	Smoking	0.41	2.18	0.01	0.19	0.851
	Consumption of tea/coffee	5.32	2.13	0.15	2.49	0.013
	Thought of changing profession	6.64	2.65	0.17	2.50	0.013
	Thought of changing specialty	2.68	2.37	0.08	1.13	0.258
	Satisfied with training/job	-7.24	2.14	-0.20	-3.38	0.001
	Number of working hours	1.63	2.40	0.05	0.68	0.496
	Single longest duty hours	4.32	2.82	0.11	1.53	0.127
**Client-related burnout**	Current training/residency status	-0.91	1.44	-0.05	-0.63	0.527
	Marital status	5.41	3.68	0.16	1.47	0.143
	Have children	0.92	3.14	0.02	0.29	0.770
	Living with parents	-0.58	2.94	-0.02	-0.20	0.845
	Have another paid employment	-1.24	2.87	-0.03	-0.43	0.665
	Financially supporting self/others	2.91	2.31	0.09	1.26	0.209
	Able to support self financially	-2.65	2.23	-0.07	-1.19	0.236
	Own a car	2.15	2.13	0.06	1.01	0.315
	Own a smartphone	-8.47	5.16	-0.10	-1.64	0.102
	Sleep duration per 24 h	-1.27	2.02	-0.04	-0.63	0.531
	Smoking	2.75	2.21	0.08	1.24	0.215
	Consumption of tea/coffee	4.00	2.14	0.11	1.87	0.063
	Thought of changing profession	8.09	2.68	0.20	3.01	0.003
	Thought of changing specialty	1.84	2.40	0.05	0.77	0.445
	Satisfied with training/job	-8.76	2.15	-0.24	-4.07	< 0.001
	Number of working hours	5.55	2.66	0.17	2.09	0.038
	Single longest duty hours	-3.54	2.85	-0.09	-1.24	0.216

## Discussion

Burnout among unmatched trainees and residents in surgical and nonsurgical specialties is a global concern [7]. Despite the considerable changes in residency programs, this phenomenon appears to be continuously escalating on a global level [7, 32]. While earlier research has focused on the general population or patients [33], little attention has been dedicated to unmatched trainees and residents in surgical and nonsurgical specialties in Palestine. In this study, the prevalence of burnout among unmatched trainees and residents in surgical and nonsurgical specialties in Palestine was determined for the first time. Additionally, factors that were associated with and predictors of higher burnout scores were also identified. In this study, the prevalence rates of moderate-severe degrees of burnout in the personal, work-related, and client-related domains were 81.2%, 75.2%, and 38.8%, respectively. Findings of this study could be informative to those in healthcare authorities, hospital managers, professional groups, directors of residency programs, and educators/trainers who might need to design appropriate interventions to address this issue and improve the well-being of unmatched trainees and residents in surgical and nonsurgical specialties.

In the personal and work-related domains, the prevalence of moderate-severe burnout among Palestinian unmatched trainees and residents in surgical and nonsurgical specialties was higher than those reported in previous studies elsewhere [7]. On the other hand, the prevalence of moderate-severe burnout among Palestinian unmatched trainees and residents in surgical and nonsurgical specialties in the client-related domain was slightly lower than those reported in previous studies elsewhere [7]. It is noteworthy mentioning that the reported prevalence rates of burnout varied significantly among professionals in different specialties and healthcare settings investigated [7, 34–38]. In this study, burnout scores were not different between unmatched trainees, residents in surgical specialties, and residents in nonsurgical specialties. The findings of this study indicate that medical training and specialty residency can be associated with stress and burnout. Results from this study were consistent with those reported among trainees and residents in different countries [39, 40]. The findings of this study should be considered when making recommendations to reduce burnout among trainees and residents in similar settings [41, 42].

Multiple previous studies have investigated the prevalence of burnout among healthcare providers in different specialties including trainees and residents in surgical and nonsurgical specialties [3, 7]. On the other hand, few studies were conducted to investigate the prevalence of burnout among trainees and residents in surgical and nonsurgical specialties in middle- and low-income countries including Arab countries [43]. In a systematic review, Elbarazi et al. included 19 studies reporting on the prevalence of burnout among healthcare providers in Arab countries [44]. Of those, only one study reported on the prevalence of burnout among a small sample of orthopedic surgeons in Saudi Arabia at 51% [45]. In Palestine, the prevalence of burnout was investigated among social workers and nurses [44]. In addition to differences in stressors among different specialties, discrepancies in prevalence rates of burnout could also be explained by cultural and religious differences, as culture and religion were believed to shape emotions [24].

In this study, married trainees and residents in surgical and nonsurgical specialties reported significantly higher personal burnout compared to their single counterparts. Probably, married professionals have more responsibilities related to a spouse, children, and other household-related financial and social burdens [3, 7]. The findings of this study contradicted those reported by West et al. in which marriage was a probable protector of developing burnout [46]. On the other hand, those who had another paid employment reported significantly less personal and work-related burnout. Previous studies reported a connection between financial stability and decreased levels of burnout [11, 47]. Probably, having another paid employment promoted a sense of financial security/stability. In this study, trainees and residents in surgical and nonsurgical specialties who reported an inability to support themselves financially reported higher work-related burnout. Trainees and residents who reported frequent consumption of tea/coffee also reported significantly higher personal and work-related burnout scores. The findings of this study were consistent with those reported among trainees and residents in other countries [43]. In different cultures, stressed individuals including healthcare providers turn to consume more tea/coffee [43, 46, 48]. In this study, trainees and residents who were dissatisfied with their training/job reported significantly higher personal, work-related, and client-related burnout. Additionally, trainees and residents who reported higher personal and work-related burnout also thought of changing their profession. These findings were not surprising as dissatisfaction with one’s training/job conditions might increase stress and burnout. The findings of this study were consistent with those reported in previous studies [3, 7, 43, 46, 48]. Longer working hours and excessive workload including overnight shifts were reported to be associated with higher burnout among residents [10–12]. Previous studies also reported that burnout contributed to increased job turnover and abandoning the residency programs [20, 21]. In this study, gender was not associated with burnout. The findings of this study contradicted those in previous studies in which female residents reported higher burnout compared to their male peers [9]. Our findings indicated that training and residency in surgical and nonsurgical specialties were equally stressful for both male and female trainees/residents.

The findings of this study provided more insights into burnout among trainees and residents in Palestine. It has been argued that gaining insights into stress and early signs of burnout may help decision-makers set policies and plan cognitive, behavioral, and mindful interventions that might be effective in minimizing burnout symptoms and improving the well-being of trainees and residents [1, 3, 7, 12, 21, 26, 47]. Our findings might highlight the need to improve the financial stability/security of married trainees and residents in surgical and nonsurgical specialties. Additionally, the findings of this study might highlight the need to improve the working conditions and training/job satisfaction of trainees and residents in Palestine.

## Strengths and limitations

The findings of this study should be interpreted considering the following strengths. This was the first study to determine the prevalence of burnout among trainees and residents in surgical and nonsurgical specialties in Palestine. The study also identified predictors of higher burnout scores across the personal, work-related, and client-related domains. In this study, a previously validated and reliable burnout inventory was used to determine the prevalence of burnout among the study participants. Although the Copenhagen Burnout Inventory was previously validated, the tool was pilot tested for stability of scores over a short time and internal consistency of the items in the different domains. The tool was shown to be reliable and internally consistent. The sample included in this study was diversified in terms of gender, place of work, and the geographical distribution across the West Bank of Palestine. The response rate was relatively high compared to other studies in which a questionnaire was used as the study tool.

On the other hand, the study has some limitations. First, this was a cross-sectional study. Cross-sectional studies are limited by design and the findings are time-bound. A larger longitudinal study should have provided more insights into how burnout syndrome evolved among trainees and residents in surgical and nonsurgical specialties. Second, a short time was allowed between the two rounds in the rest-retest method. This short time interval might have led to cognitive bias. However, the Copenhagen Burnout Inventory was previously shown to be reliable and internally consistent. Third, the answers obtained in this study are self-reported. Probably, some residents and trainees in surgical and nonsurgical specialties might have downplayed some of their burnout symptoms. Additionally, recall bias cannot be ruled out among respondents who were nearing the completion of their program. Those could have recalled their experiences more positively compared to their peers who were still going through the stresses of the early years. Third, the study was conducted during the ongoing COVID-19 pandemic. During this period, trainees and residents had to work extra shift hours due to the increasing number of patients admitted to the hospitals. The heavy workload during this period could have led to more burnout [49, 50]. Fourth, although burnout symptoms were assessed using a valid inventory, no further clinical assessments were conducted by psychologists/psychiatrists to assess burnout among the participants in this study. Finally, although the response rate was considerably high, we cannot rule out the possibility of non-response bias as “burned out” potential participants did not want to participate in the study and we did not want to disclose their burnout. Thus, potentially losing responses from a significant population.

## Conclusion

The findings of this study indicated that burnout was highly prevalent among trainees and residents in surgical and nonsurgical specialties in Palestine. Higher burnout scores were predicted by being married, not having another paid employment, inability to financially support oneself, frequent consumption of tea/coffee, dissatisfied with their training/job, thinking of changing the profession, and long working hours. Decision-makers in healthcare authorities, hospital managers, professional groups, directors of residency programs, and educators/trainers should consider addressing burnout and improving the well-being of trainees and residents in surgical and nonsurgical specialties in Palestine. Future studies are still needed to determine which interventions could be effective in reducing burnout among trainees and residents in surgical and nonsurgical specialties in Palestine.

## Supplementary Information

**Additional file 1: Supplementary Table S1: **Adherence to the guidelines of reporting of cross-sectional studies in which aquestionnaire was used as the study tool. **Supplementary Fig. S1. **Training centers/hospitals (the map was adopted and modified from WikimediaCommons that can be accessed from: http://commons.wikimedia.org/wiki). **Supplementary Table S2: **The first part of the questionnaire.

## Data Availability

All data relevant to this study were included in the manuscript or provided as supplementary materials. The datasets used and/or analyzed during the current study are available from the corresponding author on reasonable request.
