# Supplementary material for: Prevalence of burnout syndrome among unmatched trainees and residents in surgical and nonsurgical specialties: a cross-sectional study from different training centers in Palestine
Source: BMC Med Educ. 2022 Apr 26;22:322. doi: 10.1186/s12909-022-03386-8 (PMC9041277; doi:10.1186/s12909-022-03386-8)
Supplement: Supplementary file 1 — Additional file 1: Supplementary Table S1: Adherence to the guidelines of reporting of cross-sectional studies in which aquestionnaire was used as the study tool. Supplementary Fig. S1. Training centers/hospitals (the map was adopted and modified from WikimediaCommons that can be accessed from: http://commons.wikimedia.org/wiki). Supplementary Table S2: The first part of the questionnaire. [file 12909_2022_3386_MOESM1_ESM.docx]

**Supplementary materials for the manuscript:**

**Prevalence of** **burnout syndrome among unmatched trainees and residents in surgical and nonsurgical specialties: a cross-sectional study from different training centers in Palestine**

Ramzi Shawahna^1,2*^, Iyad Maqboul^3,4*^, Ola Ahmad^3^, Afnan Al-Issawy^3^, Batoul Abed^3^

^1^Department of Physiology, Pharmacology and Toxicology, Faculty of Medicine and Health Sciences, An-Najah National University, Nablus, Palestine

^2^An-Najah BioSciences Unit, Centre for Poisons Control, Chemical and Biological Analyses, An-Najah National University, Nablus, Palestine

^3^Department of Medicine, Faculty of Medicine and Health Sciences, An-Najah National University, Nablus, Palestine

^4^An-Najah National University Hospital, An-Najah National University, Nablus, Palestine

**^*^Correspondence:**

Ramzi Shawahna, PhD, Department of Physiology, Pharmacology and Toxicology, Faculty of Medicine & Health Sciences, New Campus, Building: 19, Office: 1340, An-Najah National University, P.O. Box 7, Nablus, Palestine

Iyad Maqboul, MD, Department of Medicine, Faculty of Medicine & Health Sciences, An-Najah National University, P.O. Box 7, Nablus, Palestine

Phone: + (970) 923 45113 ext 2772

Phone: + (970) 92349739

Email: [ramzi_shawahna@hotmail.com](mailto:ramzi_shawahna@hotmail.com)**Supplementary Table S1:** Adherence to the guidelines of reporting of cross-sectional studies in which a questionnaire was used as the study tool [[1-3](#_ENREF_1)]

| **Checklist Item** | **Explanation** | **Place in the manuscript** |
| --- | --- | --- |
| **Title and abstract** |  |  |
| Design of the study stated | Are the words 'questionnaire' or 'survey' stated in the title and/or abstract? | The term “questionnaire” was mentioned in the title and the abstract as well as in other sections of the manuscript |
| **Introduction/Background** |  |  |
| Background provided | Did the authors present a well-written background to their research? | A background to the study was provided under Background section in this manuscript |
| Purpose/aim of paper explicitly stated | Did the authors identify a specific purpose, aim, goal, or objective of the study? | The objectives of the study were provided in the last paragraph of the Background section |
| **Tool of measurement** |  |  |
| Description of the questionnaire | Did they provide access to the questionnaire items used in the study in either the article, appendices, or an online supplement? | Items in the questionnaire were described. References to the questionnaire were provided. |
| References to original work provided | Did they provide a reference to the existing tool (questionnaire) that they used? | References to the previous works in which the questionnaire was used were provided under (Study tool) |
| Psychometric properties | Psychometric properties = (were the reliability and psychometric of the existing questionnaire mentioned?) | Reliability and psychometric were provided under (Study tool) |
| Description of the scoring procedures provided | Did papers which used survey instruments that required scoring provide a description of the scoring procedures? | Provided under (Statistical analysis) |
| **Recruitment process and sample description** |  |  |
| Description of survey population and sample frame | Survey population = the main target of the study. Sample frame = the methods they used to reach this target. For instance, if a study wanted to detect the prevalence of hypertension in elderly people above 65 years old, and researchers went to 10 elderly care centers and randomly recruited 50 old men from the registry of each of those centers. This means that our survey population is elderly people above 65, and the sample frame is the 10 care centers registry. | Provided under (Data collection) |
| Description of representativeness of the sample | Is a description of whether the sample will represent the whole population provided? | Provided under (Study participants and sample size) |
| Sample size calculation or rationale/justification presented | Did they mention a description of their sample size calculation, such as providing a formula or a rationale? | Provided under (Study participants and sample size) |
| Incentives | Were any incentives offered (e.g., monetary, prizes, or non-monetary incentives such as an offer to provide the survey results)? | Provided under (Data collection) |
| **Survey Administration** |  |  |
| Mandatory/voluntary | Was it a mandatory survey to be filled in by every visitor who wanted to enter the Website, or was it a voluntary survey? | Provided under (Data collection) |
| Dates | In what timeframe were the data collected? | Provided under (Study context/design) |
| **Analysis** |  |  |
| Methods of data analysis | Was a description of the variables that were analyzed, how they were manipulated, and the statistical methods that were used provided? | Provided under (Statistical analysis) |
| **Results** |  |  |
| Response rate reported | Was response rate reported? | Provided under (Response rate and characteristics of the participants) |
| Results clearly presented | _ | Provided in the Results section |
| Results address objectives | _ | Provided in the Results section |
| **Discussion** |  |  |
| Results summarized referencing study objectives | _ | Provided in the first paragraph of the Discussion section |
| Strengths of the study stated | _ | Provided under Strength and Limitations |
| Limitations of the study stated | _ | Provided under Strength and Limitations |
| Generalizability of results discussed | Did they include any discussion on the generalizability of their results? | Discussed under Strength and Limitations |
| **Ethical Quality Indicators** |  |  |
| Study funding reported |  | Provided in the Declarations |
| Research Ethics Board (REB) review reported |  | Provided in the Declarations |
| Subject consent procedures reported |  | Provided in the Declarations |


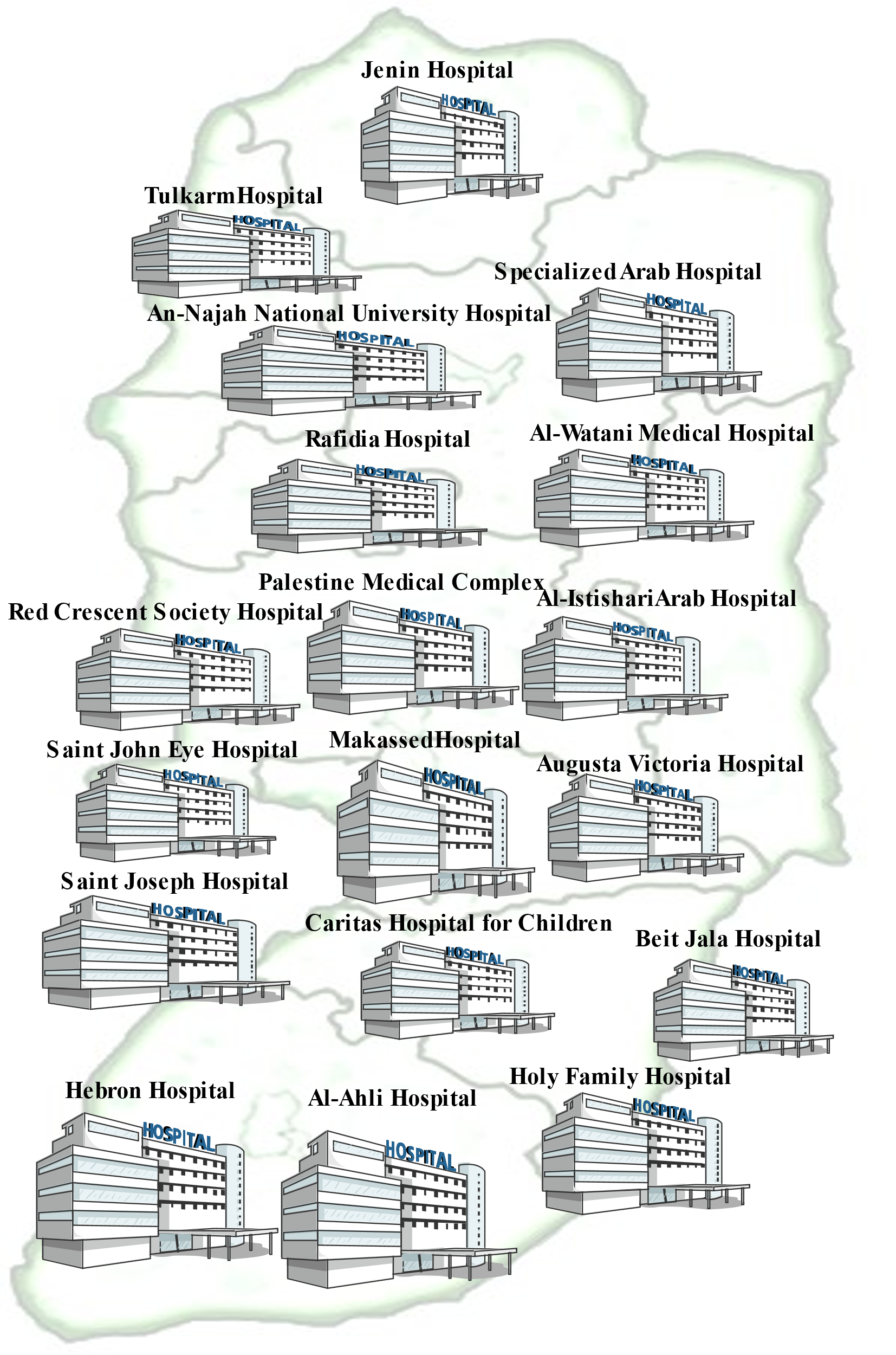


**Supplementary Fig. S1** Training centers/hospitals (the map was adopted and modified from Wikimedia Commons that can be accessed from: http://commons.wikimedia.org/wiki)

**Supplementary Table S2:** The first part of the questionnaire

| What is the current status of your training/residency? | □ Unmatched trainee | □ Resident in a surgical specialty | □ Resident in a nonsurgical specialty |
| --- | --- | --- | --- |
| Please indicate your gender | □ Male | □ Female |  |
| Please provide your current marital status | □ Single | □ Married |  |
| Do you have children? | □ No | □ Yes |  |
| Do you live with your parents? | □ No | □ Yes |  |
| Other than your current job/training at the hospital, do you have another paid employment? | □ No | □ Yes |  |
| Do you feel able to support yourself financially | □ No | □ Yes |  |
| Currently, you are financially supporting: | □ Only myself | □ Wife/children/parents |  |
| Do you own a car? | □ No | □ Yes |  |
| Do you own a laptop? | □ No | □ Yes |  |
| Do you own a smartphone? | □ No | □ Yes |  |
| Usually, how many hours of sleep do you get per 24 hours? |  |  |  |
| Do you smoke cigarettes/tobacco? | □ No | □ Yes |  |
| Usually, how many cups of tea/coffee do you consume per day? | |  |  |
| Lately, have you seriously thought of changing your profession? | □ No | □ Yes |  |
| Lately, have you seriously thought of changing your specialty? | □ No | □ Yes |  |
| Are you satisfied with your training/job | □ No | □ Yes |  |
| How many hours do you work per week? |  |  |  |
| What was the single longest duty hours you served? |  |  |  |

The second part of the questionnaire contained the Copenhagen Burnout Inventory that can be accessed from [[4](#_ENREF_4)].

**References**

1. Turk T, Elhady MT, Rashed S, Abdelkhalek M, Nasef SA, Khallaf AM, Mohammed AT, Attia AW, Adhikari P, Amin MA *et al*: **Quality of reporting web-based and non-web-based survey studies: What authors, reviewers and consumers should consider**. *PloS one* 2018, **13**(6):e0194239.

2. Sharma A, Minh Duc NT, Luu Lam Thang T, Nam NH, Ng SJ, Abbas KS, Huy NT, Marusic A, Paul CL, Kwok J *et al*: **A Consensus-Based Checklist for Reporting of Survey Studies (CROSS)**. *Journal of general internal medicine* 2021.

3. von Elm E, Altman DG, Egger M, Pocock SJ, Gotzsche PC, Vandenbroucke JP, Initiative S: **The Strengthening the Reporting of Observational Studies in Epidemiology (STROBE) statement: guidelines for reporting observational studies**. *PLoS medicine* 2007, **4**(10):e296.

4. Kristensen TS, Borritz M, Villadsen E, Christensen KB: **The Copenhagen Burnout Inventory: A new tool for the assessment of burnout**. *Work & Stress* 2005, **19**(3):192-207.
